# Supplementary material for: Smart facemask for wireless CO2 monitoring
Source: Nat Commun. 2022 Jan 10;13:72. doi: 10.1038/s41467-021-27733-3 (PMC8748626; doi:10.1038/s41467-021-27733-3)
Supplement: Supplementary file 1 — Supplementary Information [file 41467_2021_27733_MOESM1_ESM.pdf]

Supplementary Information

Smart facemask for wireless CO_2 monitoring

P. Escobedo[1,2], M.D. Fernández-Ramos[2,3], N. López-Ruiz[1,2], O. Moyano-Rodríguez[3], A. Martínez Olmos[1,2], I.M. Pérez de Vargas-Sansalvador[2,3], M.A. Carvajal[1,2,4], L.F. Capitán-Vallvey[2,3], A.J. Palma[1,2,4,*]

[1] ECsens. CITIC-UGR, Department of Electronics and Computer Technology, University of Granada, Granada, Spain

[2] Unit of Excellence in Chemistry applied to Biomedicine and the Environment of the University of Granada, Granada, Spain

[3] ECsens. Department of Analytical Chemistry, University of Granada, Granada 18071 Spain

[4] Sport and Health University Research Institute (iMUDS), University of Granada, 18071 Granada, Spain

Correspondence should be addressed to A.J.P. (email: ajpalma@ugr.es).

1. Optimization of optical sensor composition 2

2. Sensor storage lifetime: Long-term stability study 5

3. Sensing NFC tag components 7

4. NFC antenna initial calculations and design 9

5. NFC antenna characterization 10

6. Smartphone application 12

References 15

1. Optimization of optical sensor composition

As a State-of-the-Art brief revision, Supplementary Table 1 summarizes the principal analytical characteristics of representative CO_2 luminescent gas sensors found in the literature. CO_2 detection chemistry for optical sensors is generally based on plastic solid-state sensor film that works with a colour-based or fluorescence-based pH indicator, such as α-naphtholphthalein or 8-hydroxypyrene-1,3,6-trisulfonate with pKa that matches CO_2 acidity. The limitation of this design arises from the poor stability of the membranes. Due to the small number of existing fluorescent indicators, a combination of luminescent dye with non-luminescent pH indicator has been used. This system works by both primary or secondary inner-filter effect or by resonance energy transfer (RET), measuring the intensity of the luminescence, the decay time, ratiometric signals or colour. The drawback of these strategies is the limited shelf life that arises from photobleaching of the dye, evaporation of its trace water content, and degradation of the quaternary amine hydroxide base. Different solutions have been tested, such as the inclusion of ionic liquids, or bases that do not suffer from Hoffman degradation. Another possible strategy in sensors based on secondary inner-filters is to replace the luminescent dye, whose emission is modulated by the pH indicator, by an LED with the same wavelength of the dye. Another approach developed to improve the stability of sensor membrane is to replace the luminescent dye, typically a metal complex or an organic molecule, by stable inorganic phosphors. This last solution has been adopted in this study, in which the luminescence emission of an inorganic phosphor is absorbed by the basic form of the pH indicator, thus increasing the luminescence when the CO_2 concentration increases.

Regarding the approach to measure and package the sensor, the strategy used is based on the displacement of the acid-base equilibrium of a pH indicator immobilized on the membrane in the presence of CO_2. To increase the sensitivity of the sensor towards CO_2, an inert luminescent dye, whose emission band overlaps the absorption band of the pH indicator in its basic form, is co-immobilized on the membrane. In absence of CO_2, the entire indicator will be in basic form due to the presence of a quaternary ammonium hydroxide. The luminescence of the dye will be completely cancelled by a secondary internal filter mechanism. In the presence of CO_2, there will be a shift towards the acid form of the indicator with the consequent increase in luminescence. A stable inorganic phosphor is selected as luminescent material, which increases the stability and lifetime of the membrane. Additionally, an ionic liquid is included in the membrane composition to

strengthen the sensor stability and sensitivity by increasing the solubility of CO_2 in the membrane. In addition, a surfactant is included to reduce the interfacial tension and facilitate the permeation of CO_2 gas in the membrane. All these components are encapsulated in a hydrophilic hydroxypropylmethylcellulose membrane to prevent water loss over the life of the membrane.

Supplementary Table 1. Comparison of luminescence-based sensors for CO_2 gas determination found in the literature.

Sensing chemistry	Technique	LOD (ppm)/ range (%)	Response time (s)	Precision (%)	Storage lifetime (months)	Ref.
HPTS/TBP/TOAOH	LI	0-1%	4.3	-	24-36 h	[1]
HPTS/TOAOH/sol gel/TiO_2	LI-A/DLR	80	50	-	7 days/in vacuum	[2]
Ru(dpp)/Sudan III/TOAOH	LI/LT	60	20–30	1.6	3/dark	[3]
PAAChl	FI	2	20–30	-	12/4-8ºC	[4]
PtOEP/N/TOAOH	FI	0-40%	60	-	14 days/dark	[5]
$NaYF_4$:Yb/BTB/TBAOH	FI	1100	10	1%	-	[6]
GAB/aB/IL	I	2600	23	0.70	At least 120 days/ dark	[7]
CI/ETH2412/TDDMACl	A	200	-	CI	-	[8]
La_2O_2S:Eu/N/TMAOH/	C	140	9	4.41	140 days/dark	This work

HPTS: 8-hydroxypyrene-1,3,6-trisulfonate; TBP: Tributylphosphate; TOAOH: Tetraoctylammonium hydroxide; Ru-dpp): Ruthenium (II) tris (4,7-diphenyl-1,10-phenantroline); PAAChl: Chlorophyllide b derivatized polyallylamine; PtOEP: Platinum octaethylporphyrin; N: α-Naphtholphthalein; BTB: Bromothymol blue; TBAOH: Tetrabutylammonium hydroxide; GAB: Cr(III)-doped gadolinium aluminium borate; IL: Ionic liquid; aB: AzaBodipy; Cl: Carbonate ionophore; TDDMACl: Tridodecylmethylammonium chloride; TMAOH: Tetramethylammonium hydroxide; LI: Luminescent intensity, A: Absorbance, R: Ratiometric, LT: Luminescent lifetime; DLR: Dual luminophore reference; C: Colour.

CO_2 sensing membranes were deposited on 125 µm-thick polyethylene terephthalate (PET) flexible substrate of the NFC tag by spin coating. Prior to the fabrication, an optimization process of the optical sensor composition was conducted. Different parameters were studied to optimize the membrane composition. To reduce the interfacial tension and facilitate the permeation of CO_2 gas, it was necessary to add a surfactant[9].

Different surfactants in different amounts were tested: Brij 30 (3-20 µL); Tween 20 (2-20 µL); SDS (0.5-2.5 µL) and CTAB (0.5-2.5 µL). Supplementary Table 2 shows the results obtained for each surfactant considering the response time and the maximum I_{100}-I_0 value, which is the difference in the luminescence intensity when exposed to 100% CO_2 and 100% N_2. Although with SDS the I_{100}-I_0 value was higher, the response time was very slow compared with the other surfactants; with CTBA there was a solubility problem, so Tween 20 was finally selected as the optimum surfactant.

Circular membranes were deposited using the optimized cocktail having the following rheological characteristics: surface tension 47.68 dyn·cm^{-1}, viscosity 4.11 centipoise; pH 12.20. The produced membrane has a homogeneous circular shape of 1 cm diameter with an average film thickness of 8.5 ± 0.5 µm (1 SD). The limit of detection (LOD) was obtained from exponential raw experimental data using the first three points at low CO_2 concentrations, because there is a good adjustment to a straight line, and it was calculated by LOD = t_o+ 3 s_0, where t_0 is the average blank signal and s_0 is the standard deviation of the blank, derived from twelve replicate measurements. The sensor precision was evaluated at two concentrations of CO_2 (2.0 and 20.0 %) performing 15 measurements. The obtained results show an accuracy of 8.0 and 7.8% RSD for both concentrations tested.

Supplementary Table 2. Optimization of surfactant type and amount

**Parameter**	**Surfactant**			
	**Brij 30**	**Tween 20**	**SDS**	**CTAB**
Amount (µL)	5	5	1.5	2.5
I_{100}-I_0	190 ± 2	446 ± 1	499 ± 3	170 ± 2
Response time (s)	25.4 ± 0.1	14.3 ± 0.3	160.2 ± 0.5	15.1 ± 0.2

The concentration of different constituents of the membrane was studied in the following intervals, as shown in Supplementary Fig. 1: α-naphtholthalein 1.1 mg·mL^{-1} in ethanol from 100 to 600 µL; TMAOH from 30 µL to 270 µL; La_2O_2S:Eu from 0.3 mg to 10 mg and EMIM BF4, in order to get quicker responses and lower detection limit from 1 µL to 2.5 µL. The concentration of HPMC was fixed at 1% w in water, which is the minimum concentration required to form a membrane[10]. Finally, the optimum composition for the cocktail was: 3 mg La_2O_2S: Eu, 450 µL of a solution of α-naphtholthalein 1.1 mg·mL^{-1}

in ethanol, 5 µL of Tween 20, 50 µL of TMAOH $6{,}04\cdot10^{-2}$ M, 2 µL of EMIM BF4, all of them dissolved in 1 ml of HPMC 1% in purified water.

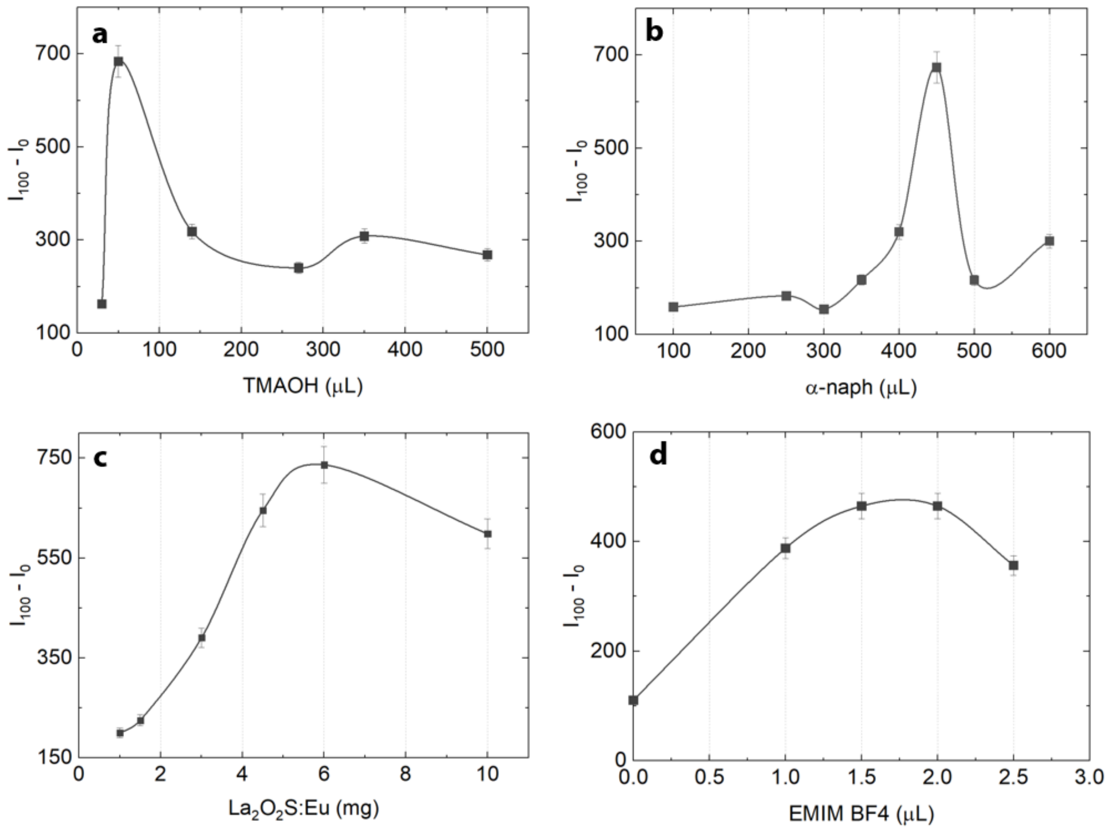

Supplementary Figure 1. Optimization of composition of CO_2 sensor. **a** Amount of TMAOH. **b** Amount of α-naphtholphthalein. **c** Amount of La_2O_2S:Eu. **d** Amount of EMIM BF4. (n=3. Data are presented as mean values +/- 1 SD).

2. Sensor storage lifetime: Long-term stability study

The long-term stability was studied keeping five sensors in darkness at room temperature, and periodically measuring the luminescence intensity, $I_{100} - I_0$ for 140 days. During this study the sensors remained stable, as depicted in Supplementary Fig. 2. As depicted in Supplementary Fig. 2, the signal intensity crossed the -2σ line on the day 140, which was considered the maximum allowed decay for the determination of the sensor lifetime. This means a percentage reduction of 6.9% in the signal intensity during the study.

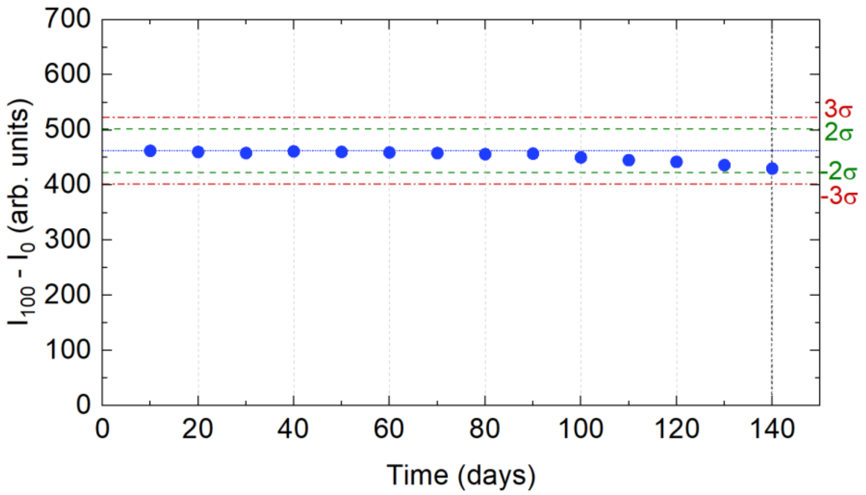

Supplementary Figure 2. Long-term stability study. Measured intensity ($I_{100} - I_0$) for 140 days. (n=5. Data are presented as mean values +/- 1 SD).

Supplementary Table 3 summarizes the principal analytical performance characteristics of the luminescent CO_2 gas sensor. The inclusion of ionic liquid improves response and recovery times as well as sensor stability. The use of inorganic luminophores improves stability, therefore sensor lifetime, and makes it possible to form a single sensor zone, which enables the development of new applications and the inclusion of portable devices[11].

Supplementary Table 3. Analytical parameters of the developed sensing membrane for CO_2 gas.

**Analytical parameter**	**CO_2 sensor**
$I_{100}-I_0$ (arb. units)	452 ± 3
Slope	3.38 ± 0.13
Intercept	0.97 ± 0.02
R^2	0.996
Measurement range (%)	0-100% CO_2
LOD (ppm)	140
Dynamic range upper limit (%CO_2)	15
Precision (DER; n = 15) at 2 % CO_2	4.41
Precision (DER; n = 15) at 20% CO_2	4.12
Response time (s)	9.00 ± 0.07
Recovery time (s)	30.40 ± 0.06
Storage lifetime (days)	140
Storage condition	In darkness

Statistical errors are one standard deviation.

3. Sensing NFC tag components

The conditioning circuitry comprises as main electronic components an extreme low-power microcontroller unit (XLP MCU) model PIC16LF1703 (Microchip Technology Inc., Arizona, USA); an AS3955 NFC Dynamic Tag IC (AMS AG, Unterpremstätten, Austria) along with a custom-designed NFC antenna; and a sensing module consisting of an ultraviolet (UV) excitation LED, a digital colour sensor and a temperature sensor. The MCU model was chosen due to its eXtreme Low-Power (XLP family) features, low operating voltage range (1.8 V to 3.6 V) and digital/analog peripherals: serial communication (I^2C and SPI), and 10-Bit Analog-to-Digital Converter (ADC). All those features were required to communicate with the NFC chip through the SPI port; with the digital colour sensor through the I^2C port; and to get the temperature readings by means of the ADC module. In fact, the MCU usage has been optimized in a way that all its available pins are used (see Fig. 3a). The AS3955 IC is an ISO14443A compliant 13.56-MHz transponder chip that allows bidirectional communication with an external NFC reader device, which in our case is the smartphone. The chip includes an advanced energy harvesting feature that enables its operation fully powered by the RF field, without any external supply. In addition, the AS3955 includes an embedded EEPROM memory that can be accessed from the PCD through the RF link or from the microcontroller through the SPI interface.

The excitation UV LED model VLMU1610-365-135 (Vishay Semiconductors, Pennsylvania, USA) is synchronised with the reading protocol of the colour detector programmed within the MCU. The UV LED was selected on the basis of the absorption spectrum of the La_2O_2S:Eu phosphor, in which the maximum peak occurs at 325 nm (see Fig. 2b). Therefore, the LED was selected to have its peak emission as close as possible to this wavelength to optically excite the CO_2 sensitive membrane, whilst meeting our design requirements in terms of low polarization voltage, low-power consumption and small size. To the best of the authors' knowledge, the selected LED model with peak emission at 367 nm was the optimum one meeting the design requirements as far as possible. Once excited by the UV LED, the luminescent response delivered by the CO_2 sensitive membrane is detected by the digital colour sensor and sent to the MCU for further processing. The digital colour sensor used in this design is the S11059-02DT (Hamamatsu Photonics, Hamamatsu, Japan), which is an I^2C interface-compatible digital detector sensitive to red (λ_{peak} = 615 nm), green (λ_{peak} = 530 nm), blue (λ_{peak} = 460 nm),

and near infrared (λ_{peak} = 855 nm) incident radiation, which is codified in the corresponding four 16-bit digital words. Its sensitivity to red emission (centred at λ=615 nm) overlaps the luminescent emission spectrum of $La_2O_2S:Eu$ for the sensing mechanism (see Fig. 2b). Besides its high resolution, this detector was selected due to its low voltage operation (2.5 V or 3.3 V) and low power consumption of only 250 μW in operation mode, which makes it optimal for our battery-less design. The colour sensor communicates with the MCU by means of an I^2C interface. A temperature sensor model MCP9700A (Microchip Technology Inc.) was also included in the design, connected to one 10-bit Analog to Digital Converter (ADC) input of the MCU, to correct the temperature drifts of the chemical sensor. This sensor was chosen due to its miniature size (2.90×1.30×0.95 mm^3) and because it presents an accuracy of ±1 °C and a very low current consumption of only 6 μA.

The temperature is calculated from the measured ADC values as per the following equation, where ADC_{data} is the digitalized value and n is the number of bits of the ADC:

$$T\ (°C) = V_{DD} \times 100 \times \frac{ADC_{data}}{2^n - 1} - 50 \qquad (1)$$

After the processing, the MCU writes the data to the EEPROM memory of the NFC chip, which is later accessed by the custom-developed smartphone application through the NFC protocol.

When the AS3955 chip is used with an appropriate antenna coil connected to the terminals LC1 and LC2, it behaves as a standard passive ISO 14443A tag, also called Proximity Inductive Coupling Card (PICC). A PICC is basically a transponder that can be read or written by a proximity reader. These tags do not have any power supply, but they are powered by the electromagnetic field induced by the NFC reader, which in our case is an NFC-enabled smartphone. The custom antenna consists of a planar coil whose inductance was designed together with the internal capacitor value (C_{RES}) of the AS3955 IC to achieve resonance at 13.56 MHz, which is the central frequency required in the NFC protocol. Considering that resonance is achieved at $f_{RES} = \frac{1}{2\pi\sqrt{L_{ANT}C_{RES}}}$, where C_{RES} = 45 pF at 13.56 MHz, the inductance value L_{RES} required for the resonance of the tag is about 3.06 μH.

A cost breakdown of the developed system is provided in Supplementary Table 4. The total price of the system is below 5 € under mass production. It is expected that the cost of the printed tag manufacturing would be only a small percentage of the total components cost under mass production. The platform cost would increase up to ~7.5 € in terms of components cost if just one unit is fabricated.

Supplementary Table 4. Cost breakdown of the developed system.

Component	Unit cost (1 unit)	Unit cost (large-scale production)
CO_2 sensitive membrane	0.002 €	0.0002 € (> 3000 units)
NFC chip	0.785 €	0.628 € (> 5000 units)
Microcontroller	0.872 €	0.695 € (> 1000 units)
Digital colour detector	3.40 €	2.498 € (> 5000 units)
UV LED	1.92 €	0.957 € (> 1000 units)
Temperature sensor	0.28 €	0.203 € (> 3000 units)
Resistors (x4)	0.085 €	0.001 € (> 3000 units)
Capacitors (x2)	0.085 €	0.004 € (> 8000 units)
**TOTAL**	**7.43 €**	**4.98 €**

4. NFC antenna initial calculations and design

The Grover Method[12] was used for the initial theoretical design of the squared planar inductor based on the following equation:

$$L_{ANT} = L_0 + \sum M \qquad (2)$$

where M is the mutual inductance between each of the inductance segments and L_0 is defined according to the following equation:

$$L_0 = \sum_{j=1}^{s} L_j \qquad (3)$$

where s is the number of segments and L_j the self-inductance of each one. According to the Grover Method, the inductance of an antenna with square coils was estimated as:

$$L_{ANT} = K_1 \mu_0 N^2 \frac{d}{1+K_2 \cdot p} \quad (4)$$

where μ_0 refers to the vacuum permeability ($4\pi \times 10^{-7}$ H/m); N is the number of turns; d is the average coil diameter defined as $d = \frac{(d_{out}+d_{in})}{2}$; p is the fill ratio calculated as $p = \frac{(d_{out}-d_{in})}{(d_{out}+d_{in})}$; and K_1 and K_2 are non-dimensional coefficients that depend on the antenna layout (e.g., square, hexagonal, octagonal, etc.). In the case of our squared-shape antenna, $K_1 = 2.34$ and $K_2 = 2.75$ [13].

5. NFC antenna characterization

Performance of NFC antennas are primarily determined by their size, quality factor (Q), capability of energy harvesting (or magnetic coupling factor), and read range. Moreover, antenna size and shape are of paramount importance for the capability of energy harvesting. It has been shown that this feature can be maximized when both the receiver and the transmitter antennas have equal dimensions[14]. This factor was firstly considered for our NFC antenna design, given the NFC antenna of the smartphone employed in this work. Taking advantage of this size, the rest of the NFC tag components were located in the inner space within the innermost antenna turn for the sake of compactness. This design strategy has been previously reported in biomedical applications[15,16].

Regarding the tag power consumption, which is 4.5 mW in our case, is comparable to other similar designs, ranging from 1.3 mW[17] to 6.5 mW[18]. In fact, according to the datasheet the selected NFC chip can provide up to 22.5 mW in optimal condition, which is higher than other NFC ICs such as SL13A (around 12 mW), MLX90129 (15 mW), and M24LR04E-R (18 mW). On the other hand, if we compare the measured Q factor = 15.15, which meets the Q factor standards of commercial coils at this resonance frequency (i.e. 13.56 MHz), is comparable to those reported previously by Rogers' group (Q~14)[19] and others[14]. Finally, the obtained read range, between 2 and 1 cm depending on the bending conditions, is also aligned with previous reports[20]. To sum up, the designed printed antenna presents features that are comparable to previous high quality NFC sensing solutions, using an affordable and compact printer compared to bulkier ink-jet and screen printers.

The frequency response of the printed antenna was evaluated using a Precision Impedance Analyzer 4294A in combination with an Impedance Probe Kit 42941A (Keysight Technologies, Santa Rosa, CA, USA). Supplementary Fig. 3a depicts the frequency response of the printed coil before attaching the NFC chip. In this case, a measured value of L_{ANT} = 3.053 μH at 13.56 MHz was obtained, which is very close to the desired inductance value. The measured quality factor at the same frequency was Q = 15.516. As shown in Supplementary Fig. 3b, after attaching the chip, thus adding the internal capacitor C_{RES} in parallel to the coil, a maximum peak of resonance was achieved at 13.72 MHz, very close to the targeted resonant frequency of 13.56 MHz.

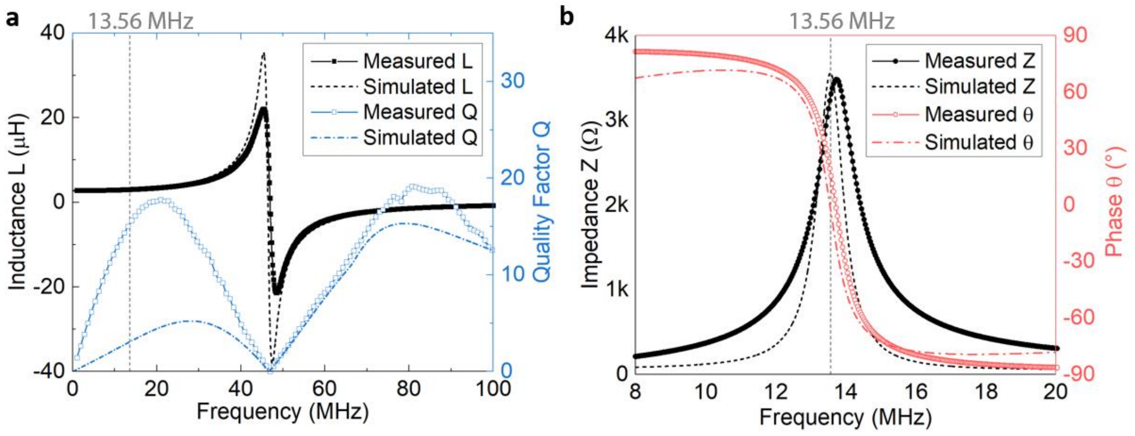

Supplementary Figure 3. Frequency response of the coil antenna. a Simulated and experimental frequency response (inductance and quality factor) of the fabricated planar inductor (n=1). **b** Simulated and experimental impedance and phase of the parallel LC circuit after attaching the NFC chip.

Regarding the exposure of the CO_2 sensor to the magnetic field induced by the NFC reader, spin chemistry teaches us that magnetic fields can influence chemical reactions that involve radical intermediates altering the rate, yield, or product distribution[21]. In this case, the chemical reactions used for CO_2 recognition are based on the modification of the acid base equilibrium position of α-naphtholphthalein and do not involve radical intermediates. In any case, according to COMSOL simulations, the NFC induced magnetic field in the tag falls into very low values, between 25 and 10 μT, without expected effect neither on the chemical sensor, nor on the electronic components.

6. Smartphone application

To enable the use of any NFC-enabled smartphone as the external reader for both data communication and powering purposes, a user-friendly custom-designed Android™ application was developed. The application takes control of the NFC interface of the smartphone to power up the tag through the electromagnetic energy from the reader's near-field link and communicates with the NFC IC. Upon approach of the smartphone, the flexible tag is immediately detected, the tag ID is shown on the smartphone screen and the firmware program within the MCU starts running. Firstly, the MCU starts the communication protocol with the digital colour sensor to acquire the measured RGB coordinates, followed by the temperature acquisition through the ADC module. Then, the MCU saves the colour and temperature measurements on specific memory locations of the NFC IC EEPROM memory. When the user clicks on the *Get Value* button through the application user interface, such locations of the EEPROM memory are accessed by the application through the ISO14443A NFC protocol. Firstly, using the obtained temperature value, the measured R coordinate is corrected to compensate for the temperature drift according to the experimental temperature dependence (see Fig. 5b). Then, the CO_2 concentration is computed from the corrected R coordinate as per the obtained calibration curve (see Fig. 5a). Finally, all the information is displayed on the smartphone screen. Moreover, the period of time wearing the facemask is automatically initiated and registered when the user clicks on the "Get Value" button for the first time. In the subsequent clicks of the button, the app shows an alert dialog window indicating the time that has passed by since the last measurement, apart from the measured CO_2 concentration. With this double information, a visual alert in the form of a traffic light indicator is proposed in a similar way of time-temperature indicators for conservation monitoring of food during transport and storage[22]. The user gets an alert regarding the air quality estimation as per the measured time-CO_2 value, showing if ventilation may be required, recommended, optional or unnecessary. Upon the recommendation, the user can restart the timer if ventilation has taken place or they can choose to continue the timer and ventilate later. In addition, the results can be shared through email and/or different cloud or messaging services using the "Share" option, thus allowing the possibility to connect to remote health care providers or medical experts. Supplementary Fig. 4 illustrates a flowchart of the developed application, showing a graphical representation

of the programmed application in relation to its sequence of functions mainly from the perspective of the user.

Supplementary Fig. 5 shows several screenshots of the custom-developed smartphone application in different scenarios. The CO_2 threshold values proposed in the traffic light indicator are purely indicative and should be taken merely as a guideline[23].

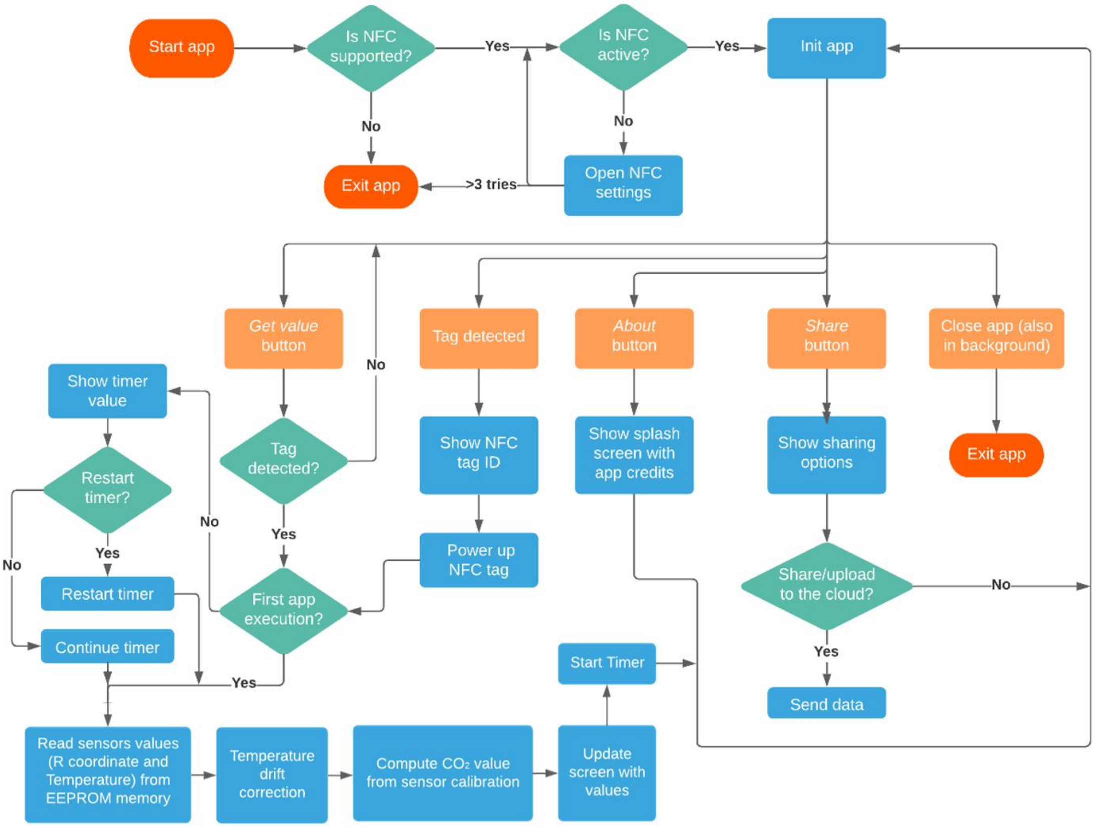

Supplementary Figure 4. Flowchart of the custom-developed smartphone application.

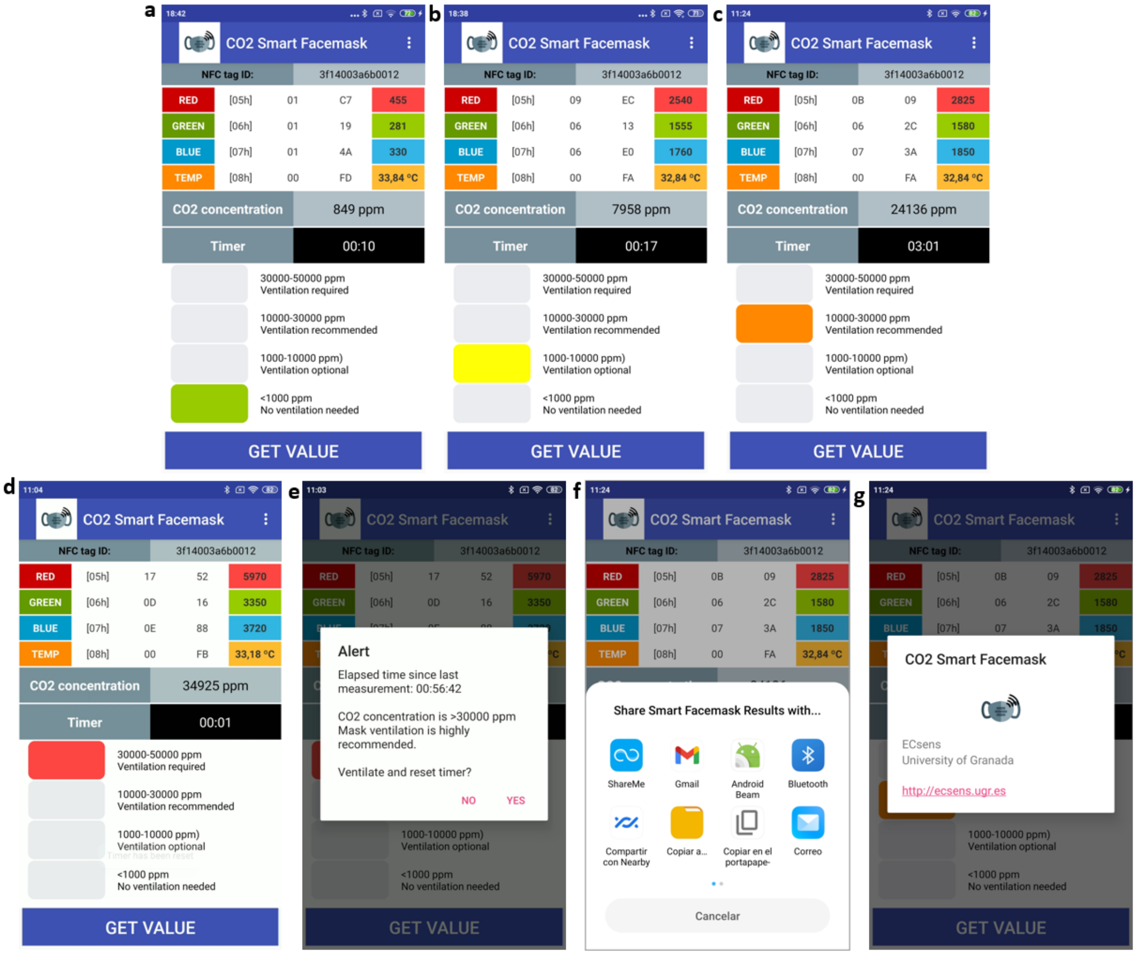

Supplementary Figure 5. a-d Screenshots of the custom-developed smartphone app showing examples of different CO_2 values and the corresponding traffic light levels as graphical indicators. **e** Example of dialog window showing an alarm with the elapsed time since the last measurement and the option to reset the timer. **f** Option “Share” where the user can send the obtained results through different messaging and/or cloud services. **f** “About” pop-up window containing the contact information.

References

1. Mills, A. & Chang, Q. Fluorescence plastic thin-film sensor for carbon dioxide. *Analyst* **118**, 839–843 (1993).
2. Dansby-Sparks, R. N. *et al.* Fluorescent-Dye-Doped Sol−Gel Sensor for Highly Sensitive Carbon Dioxide Gas Detection below Atmospheric Concentrations. *Analytical Chemistry* **82**, 593–600 (2010).
3. von Bültzingslöwen, C., McEvoy, A. K., McDonagh, C. & MacCraith, B. D. Lifetime-based optical sensor for high-level pCO_2 detection employing fluorescence resonance energy transfer. *Analytica Chimica Acta* **480**, 275–283 (2003).
4. Hamer, M., Lazaro Martinez, J. M. & Rezzano, I. N. Fluorescent responsive chlorophyllide-hydrogel for carbon dioxide detection. (2016) doi:10.1016/j.snb.2016.06.130.
5. Borchert, N. B., Kerry, J. P. & Papkovsky, D. B. A CO_2 sensor based on Pt-porphyrin dye and FRET scheme for food packaging applications. *Sensors and Actuators B: Chemical* **176**, 157–165 (2013).
6. Ali, R. *et al.* Upconverting nanoparticle based optical sensor for carbon dioxide. *Sensors and Actuators B: Chemical* **150**, 126–131 (2010).
7. Fernández-Ramos, M. D., Mirza-Montoro, F., Capitán-Vallvey, L. F. & Pérez de Vargas-Sansalvador, I. M. Near Infrared Sensor to Determine Carbon Dioxide Gas Based on Ionic Liquid. *Coatings* **11**, 163–163 (2021).
8. Xie, X., Pawlak, M., Tercier-Waeber, M.-L. & Bakker, E. Direct Optical Carbon Dioxide Sensing Based on a Polymeric Film Doped with a Selective Molecular Tweezer-Type Ionophore. *Anal. Chem.* **84**, 3163–3169 (2012).
9. El Nahhal, I. M., Zourab, S. M., Kodeh, F. S. & Qudaih, A. I. Thin film optical BTB pH sensors using sol–gel method in presence of surfactants. *International Nano Letters* **2**, 16–16 (2012).
10. Aguayo-López, M. L., Capitán-Vallvey, L. F. & Fernández-Ramos, M. D. Optical sensor for carbon dioxide gas determination, characterization and improvements. *Talanta* **126**, 196–201 (2014).
11. Fernández-Ramos, M. D. *et al.* Optical portable instrument for the determination of CO_2 in indoor environments. *Talanta* **208**, 120387–120387 (2020).
12. Greenhouse, H. Design of Planar Rectangular Microelectronic Inductors. *IEEE Transactions on Parts, Hybrids, and Packaging* **10**, 101–109 (1974).
13. ST Microelectronics. AN2866 Application Note: How to design a 13.56 MHz customized tag antenna. 1–24 (2009).
14. Cao, Z. *et al.* Near-Field Communication Sensors. *Sensors* **19**, 3947–3947 (2019).
15. Chung, H. U. *et al.* Binodal, wireless epidermal electronic systems with in-sensor analytics for neonatal intensive care. *Science* **363**, eaau0780–eaau0780 (2019).
16. Yang, S. *et al.* “Cut-and-Paste” Manufacture of Multiparametric Epidermal Sensor Systems. *Advanced Materials* **27**, 6423–6430 (2015).
17. Vennemann, B., Obrist, D. & Rösgen, T. A smartphone-enabled wireless and batteryless implantable blood flow sensor for remote monitoring of prosthetic heart valve function. *PLOS ONE* **15**, e0227372–e0227372 (2020).
18. Escobedo, P. *et al.* General-purpose passive wireless point–of–care platform based on smartphone. *Biosensors and Bioelectronics* **141**, 111360–111360 (2019).
19. Kim, J. *et al.* Miniaturized Flexible Electronic Systems with Wireless Power and Near-Field Communication Capabilities. *Advanced Functional Materials* **25**, 4761–4767 (2015).
20. Lazaro, A., Villarino, R. & Girbau, D. A Survey of NFC Sensors Based on Energy Harvesting for IoT Applications. *Sensors* **18**, 3746–3746 (2018).
21. Rodgers, C. T. Magnetic field effects in chemical systems. *Pure and Applied Chemistry* **81**, 19–43 (2009).
22. Ahvenainen, R. *Novel Food Packaging Techniques*. (Woodhead Publishing, 2003).
23. Azuma, K., Kagi, N., Yanagi, U. & Osawa, H. Effects of low-level inhalation exposure to carbon dioxide in indoor environments: A short review on human health and psychomotor performance. *Environment International* **121**, 51–56 (2018).
