# Supplementary material for: Smart facemask for wireless CO2 monitoring
Source: Nat Commun. 2022 Jan 10;13:72. doi: 10.1038/s41467-021-27733-3 (PMC8748626; doi:10.1038/s41467-021-27733-3)
Supplement: Supplementary file 3 — Description of Additional Supplementary Files [file 41467_2021_27733_MOESM3_ESM.docx]

Description of Additional Supplementary Files

Title: Supplementary Movie 1

Description: **Demo video of the smart facemask for wireless CO_2_ monitoring**

Title: Supplementary Software

Description: **Source codes for MCU firmware and Android smartphone application**
