# Supplementary figures and images for: Smart facemask for wireless CO2 monitoring
Source: Nat Commun. 2022 Jan 10;13:72. doi: 10.1038/s41467-021-27733-3 (PMC8748626; doi:10.1038/s41467-021-27733-3)

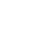

Supplement: Supplementary file 5 — Supplementary Software [file 41467_2021_27733_MOESM5_ESM.zip › SmartMaskCO2_Code/Smartphone/CO2_smartmask/app/build/generated/res/pngs/debug/drawable-hdpi/ic_info_outline_white_24dp.png]

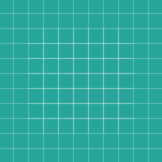

Supplement: Supplementary file 5 — Supplementary Software [file 41467_2021_27733_MOESM5_ESM.zip › SmartMaskCO2_Code/Smartphone/CO2_smartmask/app/build/generated/res/pngs/debug/drawable-hdpi/ic_launcher_background.png]

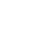

Supplement: Supplementary file 5 — Supplementary Software [file 41467_2021_27733_MOESM5_ESM.zip › SmartMaskCO2_Code/Smartphone/CO2_smartmask/app/build/generated/res/pngs/debug/drawable-hdpi/ic_share_white_24dp.png]

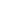

Supplement: Supplementary file 5 — Supplementary Software [file 41467_2021_27733_MOESM5_ESM.zip › SmartMaskCO2_Code/Smartphone/CO2_smartmask/app/build/generated/res/pngs/debug/drawable-ldpi/ic_info_outline_white_24dp.png]

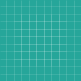

Supplement: Supplementary file 5 — Supplementary Software [file 41467_2021_27733_MOESM5_ESM.zip › SmartMaskCO2_Code/Smartphone/CO2_smartmask/app/build/generated/res/pngs/debug/drawable-ldpi/ic_launcher_background.png]

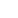

Supplement: Supplementary file 5 — Supplementary Software [file 41467_2021_27733_MOESM5_ESM.zip › SmartMaskCO2_Code/Smartphone/CO2_smartmask/app/build/generated/res/pngs/debug/drawable-ldpi/ic_share_white_24dp.png]

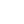

Supplement: Supplementary file 5 — Supplementary Software [file 41467_2021_27733_MOESM5_ESM.zip › SmartMaskCO2_Code/Smartphone/CO2_smartmask/app/build/generated/res/pngs/debug/drawable-mdpi/ic_info_outline_white_24dp.png]

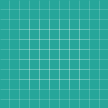

Supplement: Supplementary file 5 — Supplementary Software [file 41467_2021_27733_MOESM5_ESM.zip › SmartMaskCO2_Code/Smartphone/CO2_smartmask/app/build/generated/res/pngs/debug/drawable-mdpi/ic_launcher_background.png]

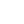

Supplement: Supplementary file 5 — Supplementary Software [file 41467_2021_27733_MOESM5_ESM.zip › SmartMaskCO2_Code/Smartphone/CO2_smartmask/app/build/generated/res/pngs/debug/drawable-mdpi/ic_share_white_24dp.png]

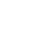

Supplement: Supplementary file 5 — Supplementary Software [file 41467_2021_27733_MOESM5_ESM.zip › SmartMaskCO2_Code/Smartphone/CO2_smartmask/app/build/generated/res/pngs/debug/drawable-xhdpi/ic_info_outline_white_24dp.png]

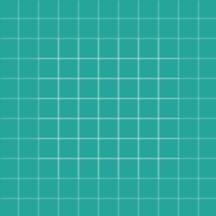

Supplement: Supplementary file 5 — Supplementary Software [file 41467_2021_27733_MOESM5_ESM.zip › SmartMaskCO2_Code/Smartphone/CO2_smartmask/app/build/generated/res/pngs/debug/drawable-xhdpi/ic_launcher_background.png]

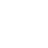

Supplement: Supplementary file 5 — Supplementary Software [file 41467_2021_27733_MOESM5_ESM.zip › SmartMaskCO2_Code/Smartphone/CO2_smartmask/app/build/generated/res/pngs/debug/drawable-xhdpi/ic_share_white_24dp.png]

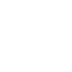

Supplement: Supplementary file 5 — Supplementary Software [file 41467_2021_27733_MOESM5_ESM.zip › SmartMaskCO2_Code/Smartphone/CO2_smartmask/app/build/generated/res/pngs/debug/drawable-xxhdpi/ic_info_outline_white_24dp.png]

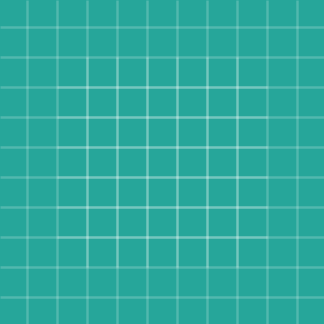

Supplement: Supplementary file 5 — Supplementary Software [file 41467_2021_27733_MOESM5_ESM.zip › SmartMaskCO2_Code/Smartphone/CO2_smartmask/app/build/generated/res/pngs/debug/drawable-xxhdpi/ic_launcher_background.png]

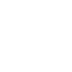

Supplement: Supplementary file 5 — Supplementary Software [file 41467_2021_27733_MOESM5_ESM.zip › SmartMaskCO2_Code/Smartphone/CO2_smartmask/app/build/generated/res/pngs/debug/drawable-xxhdpi/ic_share_white_24dp.png]

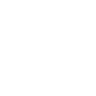

Supplement: Supplementary file 5 — Supplementary Software [file 41467_2021_27733_MOESM5_ESM.zip › SmartMaskCO2_Code/Smartphone/CO2_smartmask/app/build/generated/res/pngs/debug/drawable-xxxhdpi/ic_info_outline_white_24dp.png]

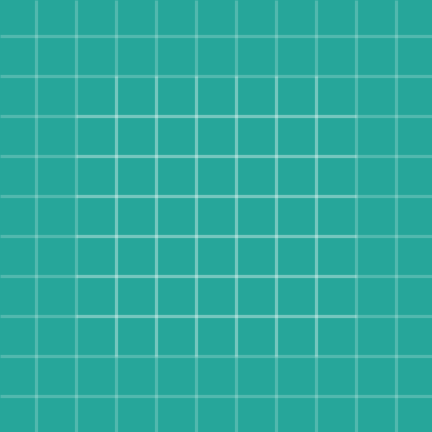

Supplement: Supplementary file 5 — Supplementary Software [file 41467_2021_27733_MOESM5_ESM.zip › SmartMaskCO2_Code/Smartphone/CO2_smartmask/app/build/generated/res/pngs/debug/drawable-xxxhdpi/ic_launcher_background.png]

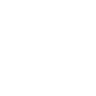

Supplement: Supplementary file 5 — Supplementary Software [file 41467_2021_27733_MOESM5_ESM.zip › SmartMaskCO2_Code/Smartphone/CO2_smartmask/app/build/generated/res/pngs/debug/drawable-xxxhdpi/ic_share_white_24dp.png]
